# Systematic implantation of dedifferentiated fat cells ameliorated monoclonal antibody 1-22-3-induced glomerulonephritis by immunosuppression with increases in TNF-stimulated gene 6

**DOI:** 10.1186/s13287-015-0069-2

**Published:** 2015-04-16

**Authors:** Takashi Maruyama, Noboru Fukuda, Taro Matsumoto, Koichiro Kano, Morito Endo, Minako Kazama, Tomohiko Kazama, Jin Ikeda, Hiroyuki Matsuda, Takahiro Ueno, Masanori Abe, Kazuyoshi Okada, Masayoshi Soma, Koichi Matsumoto, Hiroshi Kawachi

**Affiliations:** Division of Nephrology Hypertension and Endocrinology, Department of Medicine, Nihon University School of Medicine, Tokyo, Japan; Advanced Research Institute of the Sciences and Humanities, Nihon University Graduate School, Tokyo, Japan; Division of Cell Regeneration and Transplantation, Department of Functional Morphology, Nihon University School of Medicine, Tokyo, Japan; Laboratory of Cell and Tissue Biology, College of Bioresource Science, Nihon University, Fujisawa, Japan; Faculty of Human Health Science, Hachinohe Gakuin University, Hachinohe, Aomori Japan; Department of Pediatrics, Nihon University School of Medicine, Tokyo, Japan; Division of General Medicine, Department of Medicine, Nihon University School of Medicine, Tokyo, Japan; Department of Cell Biology, Institute of Nephrology, Niigata University Graduate School of Medical and Dental Sciences, Niigata, Japan

## Abstract

**Introduction:**

Implantation of mesenchymal stem cells (MSCs) has recently been reported to repair tissue injuries through anti-inflammatory and immunosuppressive effects. We established dedifferentiated fat (DFAT) cells that show identical characteristics to MSCs.

**Methods:**

We examined the effects of 10^6^ of DFAT cells infused through renal artery or tail vein on monoclonal antibody (mAb) 1-22-3-induced glomerulonephritis (as an immunological type of renal injury) and adriamycin-induced nephropathy (as a non-immunological type of renal injury) in rats. The mAb 1-22-3-injected rats were also implanted with 10^6^ of DFAT cells transfected with TSG-6 siRNA through tail vein.

**Results:**

Although DFAT cells transfused into blood circulation through the tail vein were trapped mainly in lungs without reaching the kidneys, implantation of DFAT cells reduced proteinuria and improved glomerulosclerosis and interstitial fibrosis. Implantation of DFAT cells through the tail vein significantly decreased expression of kidney injury molecule-1, collagen IV and fibronectin mRNAs, whereas nephrin mRNA expression was increased. Implantation of DFAT cells did not improve adriamycin-induced nephropathy, but significantly decreased the glomerular influx of macrophages, common leukocytes and pan T cells. However, the glomerular influx of helper T cells, was increased. Implantation of DFAT cells decreased expression of interleukin (IL)-6 and IL-12β mRNAs and increased expression of TNF-stimulated gene (TSG)-6 mRNA in renal cortex from mAb 1-22-3-injected rats. The basal level of TSG-6 protein was significantly higher in DFAT cells than in fibroblasts. Expression of TSG-6 mRNA in MCs cocultured with DFAT cells was significantly higher than in mesangial cells or DFAT cells alone. Systematic implantation of DFAT cells with TSG-6 siRNA through tail vein did not improve proteinuria, renal dysfunction and renal degeneration in the mAb 1-22-3-injected rats.

**Conclusion:**

Systematic implantation of DFAT cells effectively ameliorated mAb 1-22-3-induced glomerulonephritis through immunosuppressive effects accompanied by the suppression of macrophage infiltration and expression of IL-6, IL-10 and IL-12β, and increased production of serum and renal TSG-6 that improved the mAb 1-22-3-induced renal degeneration by the immunosuppressive effects of TSG-6. Thus DFAT cells will be suitable cell source for the treatment of immunological progressive renal diseases.

**Electronic supplementary material:**

The online version of this article (doi:10.1186/s13287-015-0069-2) contains supplementary material, which is available to authorized users.

## Introduction

Despite the availability of long-term therapies, chronic renal failure caused by immunoglobulin A (IgA) nephropathy, diabetic nephropathy and glomerulosclerosis cannot be cured through current treatments. End-stage renal disease is an appropriate application for regenerative medicine. Regarding regenerative medicines for chronic renal failure, the implantation of cells, including stem cells and progenitor cells, has been experimentally applied in treatments for progressive renal diseases [1]. To date, however, there have been no clinical trials of cell implantation for progressive renal diseases. This is because the complexity of the kidney structure prevents efficient regeneration in response to single-source cell implantation. As a source of cells for use in regenerative medicine, embryonic stem cells or inducible pluripotent stem cells possess a nearly unlimited capacity for self-renewal and have the potential to differentiate into virtually any cell type. Thus, mesenchymal stem cells (MSCs) have arisen to become a candidate cell source in regenerative medicine for kidney diseases.

Recent studies have shown that adipose tissue can provide an alternative source of MSCs [2]. Adipose tissue contains nonadipocyte cells, known as the stromal-vascular fraction, which can be isolated by centrifugation of collagenase-digested adipose tissue, which is comprised of multipotent fibroblast-like cells, known as adipose-derived stromal cells (ASCs) [3].

We established an adipogenic progenitor cell line derived from mature adipocytes and named these cells as dedifferentiated fat (DFAT) cells [4]. Clonally-expanded DFAT cells showed the ability to differentiate into multiple mesenchymal cell lineages, indicating that DFAT cells represent a type of multipotent progenitor cell. The accessibility and ease of culture of DFAT cells support their potential application to cell-based therapies [5]. In contrast to ASCs, which contain a variety of cell types, DFAT cells originate from a fraction of highly homogeneous mature adipocytes. This property of DFAT cells will likely lead to higher safety and efficacy for clinical cell therapies.

To evaluate the efficiency of cell therapy for progressive renal diseases, animal models of sustained renal failure are required. Proteinuria was maintained at a higher level and blood urea nitrogen (BUN) and serum creatinine levels were higher in rats with unilateral nephrectomy, after the administration of Thy-1.1 monoclonal antibody (mAb) 1-22-3. Morphologically, typical sclerotic changes were observed in the mAb 1-22-3 injected rats. These findings suggest that the renal lesions in the mAb 1-22-3 rats provide a suitable model for chronic progressive glomerulonephritis [6].

Implantation of MSCs has recently been reported to repair tissue injuries through their anti-inflammatory and immunosuppressive effects [7]. Implantation of MSCs has been reported to suppress fibrosis of infarcted heart [8], bleomycin-induced lung fibrosis [9], liver fibrosis [10] and interstitial fibrosis of kidney [11]. Moreover, MSCs have been established to have immunosuppressive effects. Systemic infusion of MSCs has been reported to suppress graft rejection in animal models, which has led to a number of clinical trials [12,13]. Implantation of MSCs has been developed in a clinical study in which MSCs were reported to effectively inhibit graft-versus-host disease in Phase II studies [14].

DFAT cells have the potential to differentiate into lineages of mesenchymal tissue and the cell surface antigen profile of DFAT cells has been shown to be very similar to that of bone marrow MSCs [5]. We previously examined the effects of implantation of DFAT cells on habu snake venom-induced chronic renal dysfunction in mice and found improvement of glomerulosclerosis [15]. Thus, DFAT cells may provide a source of cell therapy for severe progressive renal diseases. In the present study, to evaluate the ability of DFAT cells to serve as a cell source for progressive renal diseases, we examined the effects of implantation of DFAT cells in mAb 1-22-3-induced glomerulonephritis (as an immunological type of renal injury) and adriamycin-induced nephropathy (as a non-immunological type of renal injury) in a rat model.

## Methods

### Ethical considerations

Our investigation conformed to the Guide for the *Care and Use of Laboratory Animals*: Eighth Edition Washington, DC: The National Academies Press, 2011. The ethics committee of Nihon University School of Medicine approved all research protocols involving the use of living animals.

### Antibodies

A hybridoma producing mouse anti-rat Thy 1.1 mAb 1-22-3 (IgG3) was prepared by immunization of BALB/c mice with collagenase-treated fresh rat glomeruli. Ascitic fluid containing mAb 1-22-3 was produced in BALB/c mice primed with 2,6,10,14-tetramethylpentadecane (Sigma Chemical, St. Louis, MO, USA) and injected intraperitoneally with the hybridoma. The obtained fluid was subjected to 50% ammonium sulfate precipitation and the obtained immunoglobulin-rich fraction was dialyzed against phosphate-buffered saline (PBS).

### Preparation of the DFAT cells from adipose tissue

Approximately 1 g of subcutaneous adipose tissue from male Wistar rats was treated with collagenase and centrifuged. Adipocytes were isolated from the top layer. More than 99% of the isolated cells were mature lipid-filled adipocytes. The mature adipocytes floating on top of the culture medium attached to the upper surface of the culture flasks within a few days. Approximately 10% to 20% of the adherent cells flattened out by day 3 and changed to a spindle-shaped morphology by day 7. The cells subsequently entered a proliferative log-phase upon inversion of the flasks and changing of the media, and reached confluence by day 14. During this stage, the cells lose the lipid droplets completely and exhibit the fibroblast-like morphology of DFAT cells.

### Distribution of DFAT cells

DFAT cells from Wistar rats were labeled with a Qtracker® Cell Labeling Kit (Molecular probes, Life Technology, Tokyo, Japan). A total 10^6^ labeled DFAT cells was injected through the renal arteries or tail vein in Wistar rats. One week after the injection, kidney, aorta, liver and lungs were removed and fixed in 3% formalin in PBS and embedded in paraffin. Sections were observed under a fluorescence microscope. Images were obtained with a digital imaging system.

### Experimental protocols

**Figure 1:**
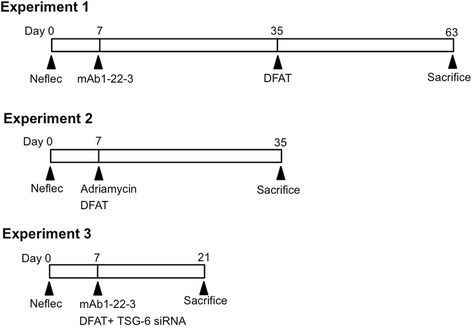
Experimental procedures. Experiment 1: Effects of implantation of DFAT cells on mAb 1-22-3-induced nephritis. In Wistar rats weighing 250 g, the right kidney was nephrectomized (Neflec). Rats were injected with 0.5 mg mAb 1-22-3 through the tail vein seven days after nephrectomy. Thirty-five days after nephrectomy, 1.0 mL saline or 10^6^ DFAT cells were injected through the renal artery or tail vein. Sixty-three days after nephrectomy, all rats were killed and the left kidney was removed. Experiment 2: Effects of implantation of DFAT cells on adriamycin-induced nephropathy. In all male Wistar rats, the right kidney was nephrectomized. Seven days after nephrectomy, rats were injected with 4 mg/kg body weight adriamycin through the tail vein and 10^6^ DFAT cells were injected through the renal artery or through the tail vein. Thirty-five days after the nephrectomy all rats were killed and the left kidney was removed. Experiment 3: DFAT cells (2 x 10^5^ cells) from Wistar rats were transfected with 20 nM rat TSG-6 siRNA or 20 nM control siRNA in siRNA Transfection Medium. In male Wistar rats weighing 250 g, the right kidney was nephrectomized. Rats were injected with 0.5 mg mAb 1-22-3 through the tail vein and 10^6^ DFAT cells transfected with TSG-6 siRNA or control siRNA were injected through the tail vein. Twenty-one days after the nephrectomy, all rats were killed and the left kidney was removed. DFAT, dedifferentiated fat; mAb, monoclonal antibody; TSG, TNF-stimulated gene.

Experiment 1: Effects of implantation of DFAT cells on mAb 1-22-3-induced nephritis. In all male Wistar rats weighing 250 g, the right kidney was nephrectomized. Control rats were observed without any further injections. Other rats were injected with 1.0 mL saline containing 0.5 mg mAb 1-22-3 through the tail vein at seven days after nephrectomy. Thirty-five days after the nephrectomy, 1.0 mL saline (Saline) or 10^6^ DFAT cells in 1.0 mL saline were injected through the renal artery (DFAT ia) or through the tail vein (DFAT iv). Sixty-three days after the nephrectomy, all rats were killed and the left kidney was removed (Figure 1).

Experiment 2: Effects of implantation of DFAT cells on adriamycin-induced nephropathy. In all male Wistar rats weighing 250 g, the right kidney was nephrectomized. Control rats were observed without any further injections. Other rats were injected with 1.0 mL saline containing 4 mg/kg body weight adriamycin (Wako Junyaku Inc. Tokyo, Japan) through the tail vein at seven days after nephrectomy without DFAT cells (Saline) or with 10^6^ DFAT cells in 1.0 mL saline. Rats were injected through the renal artery (DFAT ia) or through the tail vein (DFAT iv). Thirty-five days after the nephrectomy, all rats were killed and the left kidney was removed (Figure 1).

Experiment 3: DFAT cells (2 x 10^5^ cells) from Wistar rats were transfected with 20 nM rat TNF-stimulated gene (TSG)-6-small interfering (si) RNA or 20 nM control siRNA purchased from Santa Cruz Biotechnology, Santa Cruz, CA, USA in siRNA Transfection Medium (Santa Cruz). To confirm sufficient inhibition of expression of TSG-6 protein, we performed ELISA analysis for siRNA transfected DFAT cells 24 hours after transfection. In male Wistar rats weighing 250 g, the right kidney was nephrectomized. Rats were injected with 0.5 mg mAb 1-22-3 and 10^6^ DFAT cells transfected with TSG-6 siRNA or control siRNA through the tail vein. Fourteen days after the injections, all rats were killed and the left kidney was removed (Figure 1). Urinary protein excretion was determined with a Bio-Rad protein assay kit (Bio-Rad, Hercules, CA, USA). Serum BUN and creatinine were measured by SRL Inc. (Wako, Saitama, Japan).

### Morphological and immunohistological analysis

The 3-mm paraffin sections of removed renal cortex were stained with hematoxylin and eosin (H & E). Renal cortical thickness was measured under high magnification (×400). The glomerular injury score (GIS) was obtained using the following formula: ((0 × n0) + (1 × n1) + (2 × n2) + (3 × n3) + (4 × n4))/50. To semi-quantify the tubulointerstitial area, 20 areas of renal cortex were randomly selected. The percentage of each area that showed sclerofibrotic change was estimated and assigned a score of 0, normal; 1, involvement of <10% of the area; 2, involvement of 10% to 30% of the area; 3, involvement of 30% to 50% of the area; or 4, involvement of 50% of the area. The tubulointerstitial injury score (TIS) was calculated as ((0 × n0) + (1 × n1) + (2 × n2) + (3 × n3) + (4 × n4))/20.

Deparaffinized 5-μm sections were briefly incubated with 3% H_2_O_2_ and then with primary antibody for 60 minutes, rinsed with Tris-buffered saline containing 0.1% Tween 20, and incubated with a secondary antibody for 30 minutes. The primary antibodies used for immunohistochemical analysis were as follows: ED1 (IgG1) recognizing pan monocytes/macrophages was purchased from Chemicon International Inc. (Temecula, CA, USA); OX1 recognizing CD45^+^ common leukocytes was purchased from Santa Cruz Biotechnology (Dallas, TX, USA); Mouse anti-rat monoclonal antibody OX19 (IgG1) recognizing rat CD5 antigen was used to detect and deplete pan T cells [16]; OX38 (IgG2a) recognizing CD4^+^ helper T cells was used to detect and deplete CD4^+^ T cells [17]. OX-19 and OX-38 were precipitated from ascites using the corresponding hybridoma (European Collection of Animal Cells, Porton Down, Salisbury, UK). Counterstaining was then performed before examination under a light microscope.

### Determination of mRNA expression

Total RNA was extracted from renal cortices and cultured cells with TRIzol reagent (Life Technologies) according to the manufacturer’s instructions. Total RNA (1 μg) was reverse transcribed into cDNA with random 9-mers with a Takara RNA PCR Kit (AMV) Ver. 3.0 (Takara Bio, Ohtsu, Japan). Real-time quantitative PCR was performed with diluted cDNA using a FastStart TaqMan Probe Master (Roche Applied Science, Basel, Switzerland) and an SYBR Select Master Mix (Life Technologies) in an ABI 7500 sequence detector (Life Technologies) according to the manufacturer’s instructions. All assay-on-demand primers and probes (Kim-1, collagen IV, fibronectin, nephrin, TSG-6, TNF-α, IL-6, IL-10, IL-12β and TGF-β1) were purchased from Life Technologies. Real-time PCR data were analyzed with standard curves and normalized to 18S ribosomal RNA with its specific primer sets (5’ and 3’ primers: 5’-CGGCTACCACATCCAAGGAA-3’ and 5’-GCTGGAATTACCGCGGCT-3’) as described previously [18]. Correlation coefficients for the standard curves were all >0.90.

### Enzyme-linked immunosorbent assay

Levels of TNF-stimulated gene-6 (TSG-6) in culture medium were determined using ELISA kits (R&D Systems, Minneapolis, MN, USA). Conditioned medium for 10^6^ DFAT cells or 10^6^ fibroblasts was collected for 24 hours incubation. The supernatants of all specimens were detected by a multidetection microplate reader using a double-antibody sandwich ELISA kit according to the manufacturer’s protocols. The concentrations of TSG-6 were normalized to the total protein content.

### Coculture of DFAT and mesangial cells

Glomeruli were isolated from kidneys of four-week-old male stroke prone spontaneous hypertensive (SHR-SP) rats. Glomeruli were isolated with a graded-sieve technique as described previously [19]. Mesangial cells (MCs) from SHR-SP were plated at 4,000 cells per cm^2^ in the bottom well of a six-well transwell coculture system (Corning Incorporated, Corning, NY, USA). DFAT cells from Wistar rats were plated separately at 1,000 cells per cm^2^ on the upper inserts of six-well transwell coculture system and cultured in RPMI + 20% fetal bovine serum (FBS) for seven days. Levels of TSG-6 in culture medium were determined using ELISA. RNA was extracted from MCs from SHR-SP or DFAT cells (RNeasy Mini Kit; Qiagen, Valencia, CA, USA) and assayed for TSG-6 by real-time quantitative PCR.

### Statistical analysis

The values are reported as the mean ± SE. Student’s *t-*test was used for unpaired data. Two-way analysis of variance (ANOVA) with the Bonferroni/Dunn procedure as post-test was also used. *P* <0.05 was considered to be statistically significant.

## Results

### Distribution of implanted DFAT cells

**Figure 2:**
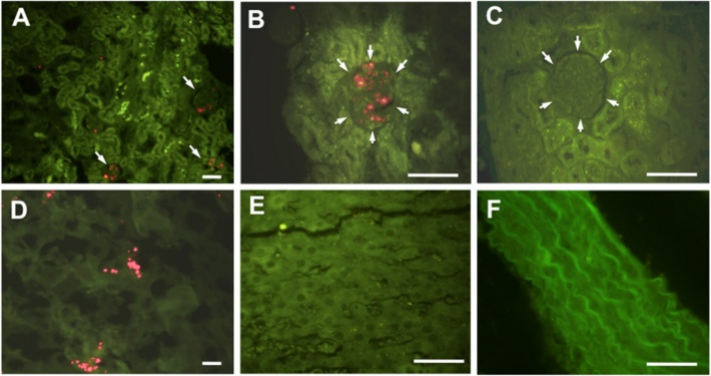
Distribution of implanted DFAT cells. DFAT cells from Wistar rats were labeled with Qtracker. A total of 10^6^ labeled DFAT cells was injected through the renal artery or tail vein in Wistar rats. One week after the injection, kidney, aorta, liver and lung were removed and fixed in 3% formalin. Renal cortex after injections of DFAT cells through renal artery **(A, B)** and through tail vein **(C)**. Arrows indicate glomerulus. Lung **(D)**, liver **(E)** and aorta **(F)**, seven days after injection of DFAT cells through the tail vein. Bar = 50 μm. DFAT, dedifferentiated fat.

A total 10^6^ Qtracker®-labeled DFAT cells was implanted through the renal artery or tail vein in Wistar rats. Most of the DFAT cells implanted through the renal artery were trapped in the glomerulus seven days after injection (Figure 2A, 2B). Seven days after injection, DFAT cells implanted through the tail vein were not delivered to the kidney (Figure 2C). They were trapped mainly in the lung (Figure 2D), but not in the liver (Figure 2E) or aorta (Figure 2F).

### Effects of implantation of DFAT cells on mAb 1-22-3-induced glomerulonephritis

**Figure 3:**
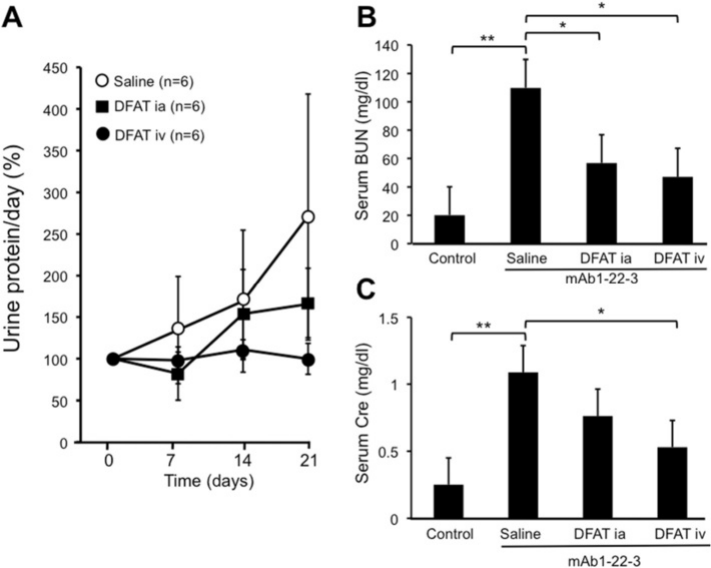
Effects of implantation of DFAT cells on proteinuria and renal function in mAb 1-22-3-induced glomerulonephritis. Wistar rats were nephrectomized and injected without mAb 1-22-3 (Control) or with 0.5 mg mAb 1-22-3 through the tail vein seven days after the nephrectomy. Thirty-five days after the nephrectomy, saline (Saline) or 10^6^ DFAT cells were injected through the renal artery (DFAT ia) or tail vein (DFAT iv). Sixty-three days after the nephrectomy, the left kidney was removed. Urinary protein excretion **(A)**, and serum levels of BUN **(B)** and creatinine (Cre) **(C)** were measured. Data are the mean ± SEM (n = 6). *P <0.05 and **P <0.01 in the indicated columns. BUN, blood urea nitrogen; DFAT, dedifferentiated fat; mAb, monoclonal antibody; SEM, standard error of the mean.

Injection of mAb 1-22-3 continuously increased proteinuria in rats. Implantation of DFAT cells through the renal artery and tail vein reduced the increases in proteinuria (not statistically significant). Systematic implantation of DFAT cells through the tail vein showed the greatest reduction of proteinuria (Figure 3A). Injection of mAb 1-22-3 significantly (*P* <0.01) increased serum levels of BUN and creatinine. Implantation of DFAT cells through the renal artery and tail vein significantly (*P* <0.05) decreased the increased serum levels of BUN and creatinine (Figure 3B, 3C).

**Figure 4:**
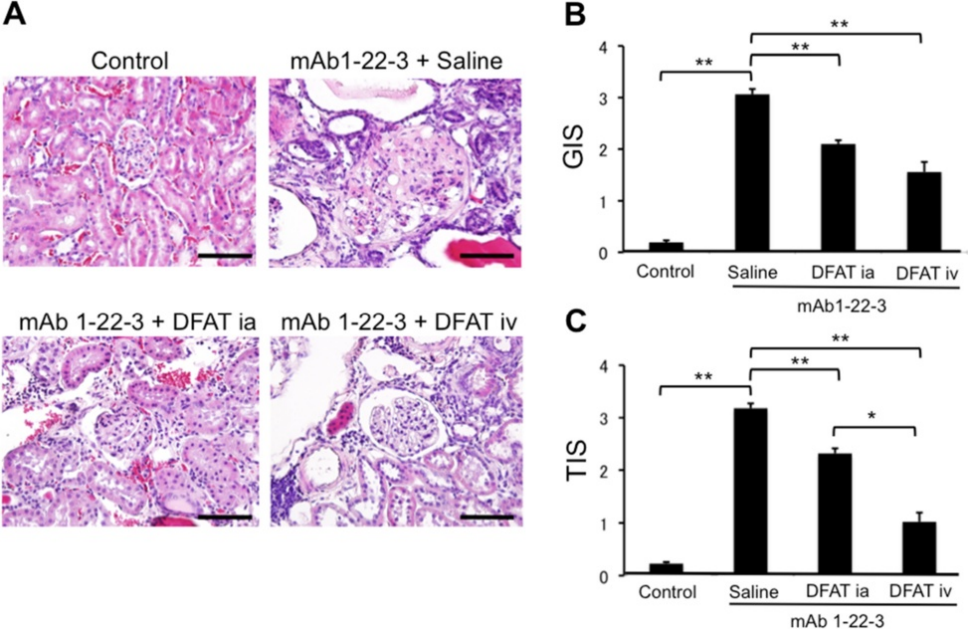
Effects of implantation of DFAT cells on degeneration of renal cortex in mAb 1-22-3-induced glomerulonephritis. Wistar rats were nephrectomized and injected without mAb 1-22-3 (Control) or with 0.5 mg mAb 1-22-3 through the tail vein seven days after the nephrectomy. Thirty-five days after the nephrectomy, saline (Saline) or 10^6^ DFAT cells were injected through the renal artery (DFAT ia) or tail vein (DFAT iv). Sixty-three days after the nephrectomy, the left kidney was removed. **A)** The paraffin sections of the removed renal cortex were stained with hematoxylin and eosin (H & E). Renal cortical thickness was measured under high magnification (x400). **B)** Glomerular injury score (GIS). **C)** Tubulointerstitial injury score (TIS). Data are the mean ± SEM (n = 6). *P <0.05 and **P <0.01 in the indicated columns. Bar = 50 μm. DFAT, dedifferentiated fat; mAb, monoclonal antibody; SEM, standard error of the mean.

Histologically, renal cortex showed sclerosis of the glomerulus, interstitial fibrosis with mesangium proliferation and infiltration of inflammatory cells two months after injection of mAb 1-22-3 (Figure 4A). Injection of mAb 1-22-3 significantly (*P* <0.01) increased the GIS and TIS. Implantation of DFAT cells through the renal artery and tail vein resulted in a significant (*P* <0.01) reduction in the increased GIS (Figure 4B) and TIS (Figure 4C). It is notable that systemic implantation, rather than direct implantation, improved glomerulosclerosis and interstitial fibrosis.

**Figure 5:**
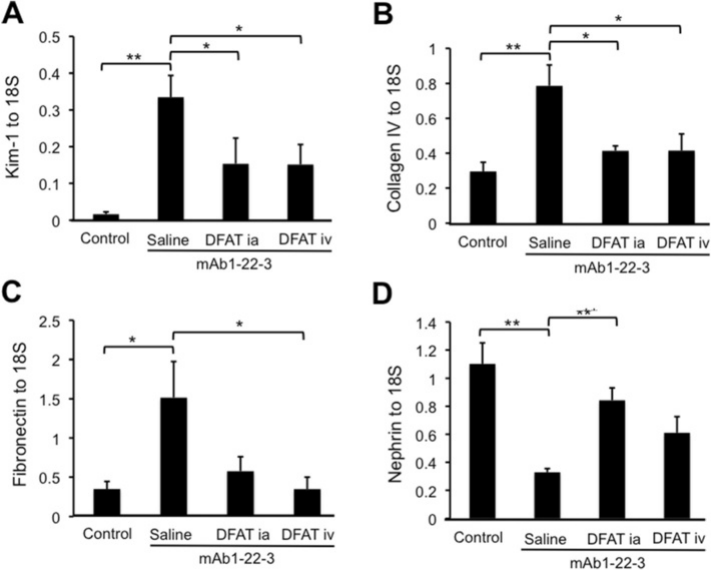
Effects of implantation of DFAT cells on mRNA expression of renal injury markers and nephrin in renal cortex in mAb 1-22-3-injected rats. Wistar rats were nephrectomized and injected without mAb 1-22-3 (Control) or with 0.5 mg mAb 1-22-3 through the tail vein seven days after the nephrectomy. Thirty five days after the nephrectomy saline (Saline) or 10^6^ DFAT cells were injected through the renal artery (DFAT ia) or tail vein (DFAT iv). Sixty-three days after the nephrectomy, the left kidney was removed. Real-time quantitative PCR was performed to determine the expression of Kim-1 **(A)**, collagen IV **(B)**, fibronectin **(C)** and nephrin **(D)** mRNAs. Data are the mean ± SEM (n = 6). *P <0.05 and **P <0.01 in the indicated columns. DFAT, dedifferentiated fat; Kim-1, kidney injury molecule; mAb, monoclonal antibody; SEM, standard error of the mean.

Figure 5 shows the effects of implantation of DFAT cells on mRNA expression of renal injury markers and nephrin in renal cortex from mAb 1-22-3-injected rats. mAb 1-22-3-injection significantly (*P* <0.05) increased the abundance of kidney injury molecule (Kim)-1, collagen IV and fibronectin mRNAs. Direct implantation significantly (*P* <0.05) decreased the abundance of Kim-1 (Figure 5A) and collagen IV mRNAs (Figure 5B). Systematic implantation significantly (*P* <0.05) decreased the abundance of Kim-1, collagen IV and fibronectin mRNAs (Figure 5A-5C). mAb 1-22-3-injection significantly (*P* <0.01) decreased the abundance of nephrin mRNA, which was significantly (*P* <0.01) increased by direct implantation (Figure 5D).

### Effects of implantation of DFAT cells on adriamycin-induced nephropathy

**Figure 6:**
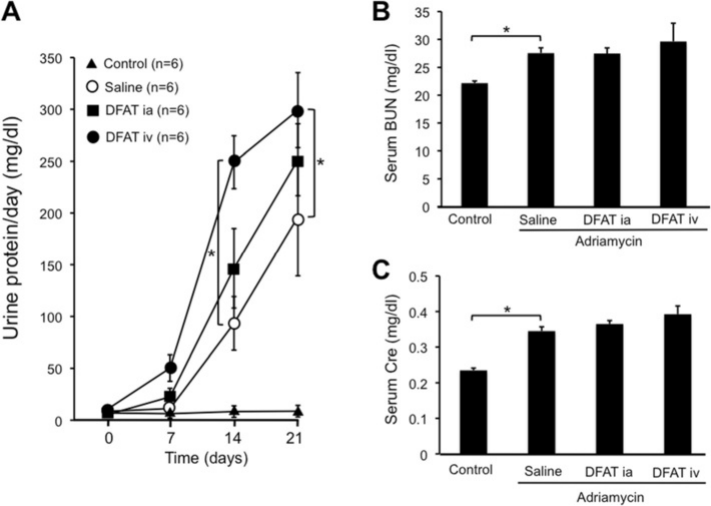
Effects of implantation of DFAT cells on proteinuria and renal function in adriamycin-induced nephropathy. Wistar rats were nephrectomized and were injected without adriamycin (Control) or with 4 mg/kg body weight adriamycin through the tail vein without DFAT cells (Saline) or with 10^6^ DFAT cells that were injected through the renal artery (DFAT ia) or tail vein (DFAT iv) in rats after the nephrectomy. Urinary protein excretion **(A)** and serum levels of BUN **(B)** and creatinine (Cre) **(C)** were measured. Data are the mean ± SEM (n = 4). *P <0.05 and **P <0.01 in the indicated columns. BUN, blood urea nitrogen; DFAT, dedifferentiated fat; SEM, standad error of the mean.

Injection of adriamycin continuously increased proteinuria in rats (Figure 6A). Systematic implantation of DFAT cells significantly (*P* <0.05) increased proteinuria compared to control (Figure 6A). Injection of adriamycin significantly (*P* <0.05) increased serum levels of BUN and creatinine. Implantation of DFAT cells did not affect serum levels of BUN and creatinine (Figure 6B, 6C).

**Figure 7:**
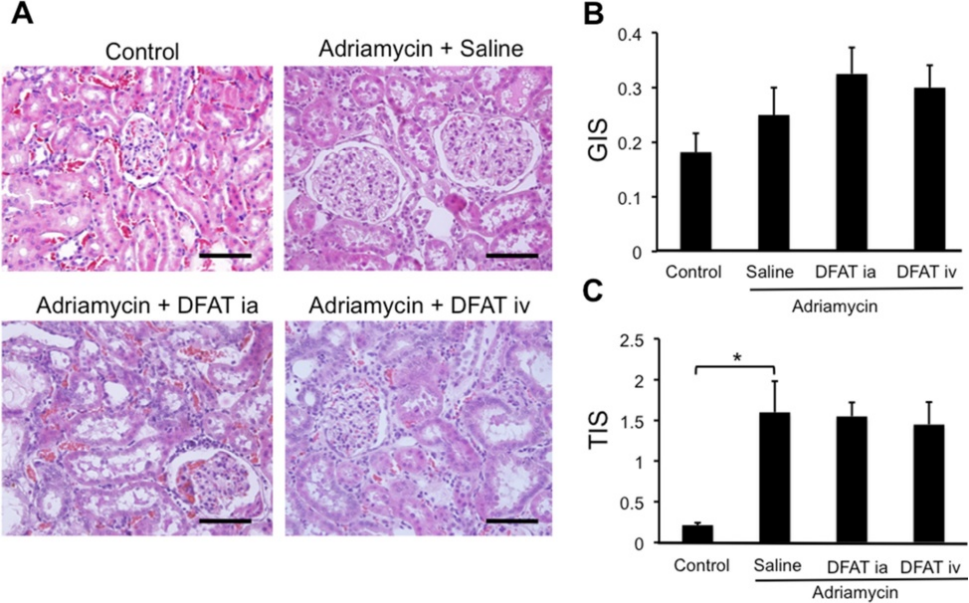
Effects of implantation of DFAT cells on degeneration of renal cortex in adriamycin-induced nephropathy. Wistar rats were nephrectomized and were injected without adriamycin (Control) or with 4 mg/kg BW adriamycin through the tail vein without DFAT cells (Saline) or with 10^6^ DFAT cells injected through the renal artery (DFAT ia) or tail vein (DFAT iv) after nephrectomy. **A)** The paraffin sections of removed renal cortex were stained with hematoxylin and eosin (H & E). Renal cortical thickness was measured under high magnification (x400). **B)** Glomerular injury score (GIS). **C)** Tubulointerstitial injury score (TIS). Data are the mean ± SEM (n = 4). *P < 0.05 and **P <0.01 in the indicated columns. Bar = 50 μm. BW, body weight; DFAT, dedifferentiated fat; SEM, standard error of the mean.

Figure 7 shows the effects of the implantation of DFAT cells on adriamycin-induced degeneration of renal cortex. Injection of adriamycin induced glomerulosclerosis and interstitial fibrosis with a significant (*P* <0.01) increase in TIS (Figure 7C). Implantation of DFAT cells did not affect adriamycin-induced glomerulosclerosis and interstitial fibrosis (Figure 7A-7C).

### Effects of implantation of DFAT cells on leukocyte accumulation in glomeruli in mAb 1-22-3-injected rats

**Figure 8:**
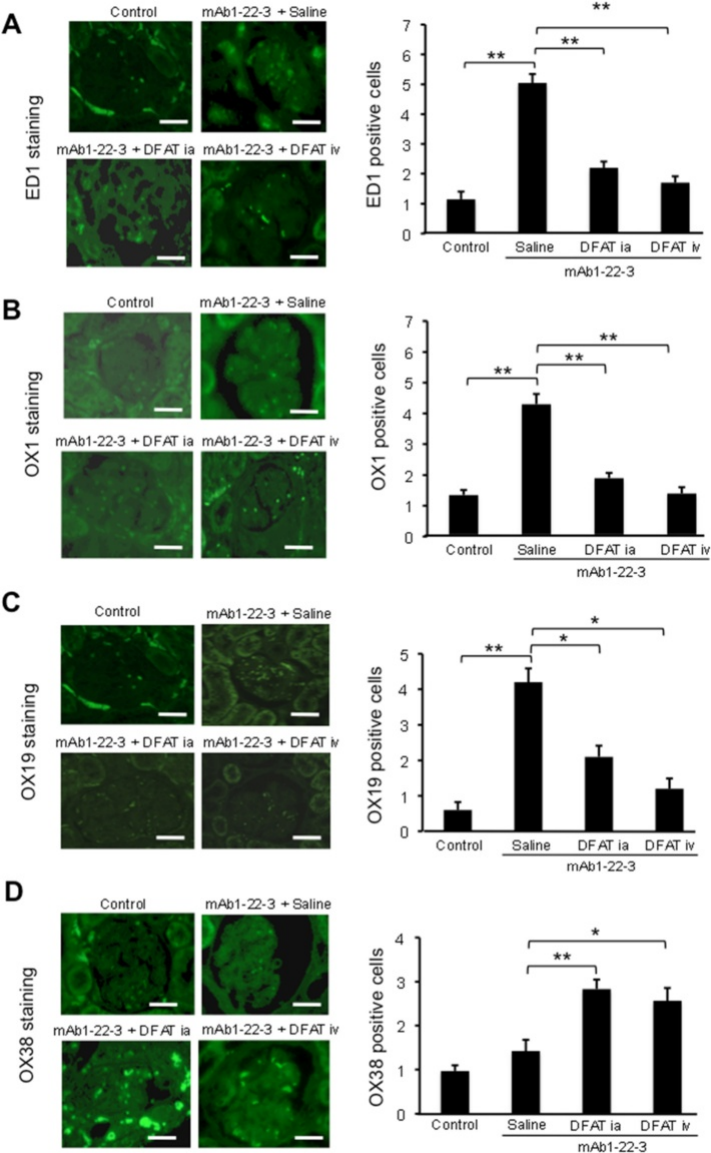
Effects of implantation of DFAT cells on leukocyte accumulation in glomeruli in mAb 1-22-3-injected rats. Wistar rats were nephrectomized and injected without mAb 1-22-3 (Control) or with 0.5 mg mAb 1-22-3 through the tail vein seven days after nephrectomy. Thirty-five days after nephrectomy, saline (Saline) or 10^6^ DFAT cells were injected through the renal artery (DFAT ia) or tail vein (DFAT iv). Sixty-three days after nephrectomy, the left kidney was removed. Immunohistochemical analysis was performed. Numbers of ED1 cells (**A**: monocytes/macrophages), OX1+ cells (**B**: common leukocytes), OX19+ cells (**C**: pan T cells) and OX38+ cells (**D**: CD4 T lymphocytes) per glomerular cross-section were counted in 50 randomly selected full-sized glomeruli. Data are the mean ± SEM (n = 6). *P <0.05 and **P <0.01 in the indicated columns. Bar = 50 μm. DFAT, dedifferentiated fat; mAb, monoclonal antibody; SEM, standard error of the mean.

To explore the immunocytological mechanisms of the effects of the implantation of DFAT cells, we evaluated leukocyte influx into the glomeruli in mAb 1-22-3-injected rats. Immunohistochemical staining of glomeruli shows significant (*P* <0.01) increases in glomerular influx of ED1^+^ cells (pan monocytes/macrophages) (Figure 8A), OX1^+^ cells (common leukocytes) (Figure 8B) and OX19^+^ cells (pan T cells) (Figure 8C) in renal cortex from mAb 1-22-3-injected rats. Implantation of DFAT cells through the renal artery and tail vein significantly (*P* <0.05) decreased the glomerular influx of ED1^+^, OX1^+^ and OX19^+^ cells (Figure 8A-8C). Implantation of DFAT cells significantly (*P* <0.05) increased the glomerular influx of OX38^+^ cells (helper T cells) (Figure 8D).

### Effects of implantation of DFAT cells on expression of cytokines in renal cortex in mAb 1-22-3-injected rats

**Figure 9:**
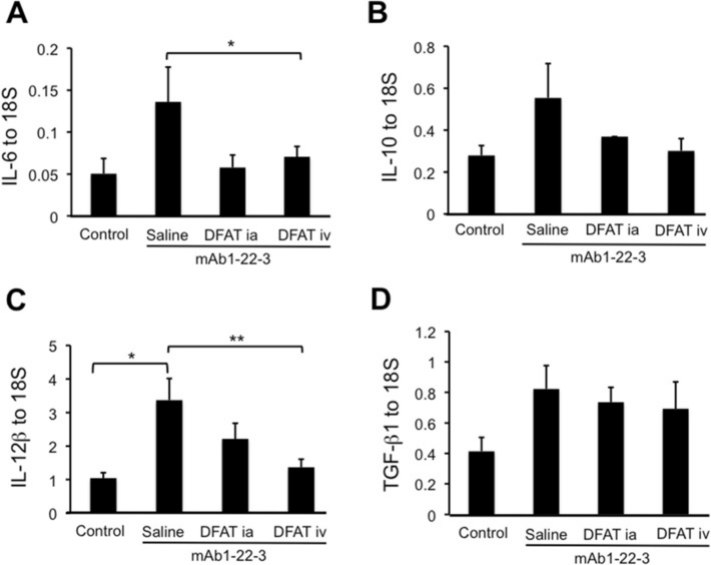
Effects of implantation of DFAT cells on mRNA expression of cytokines in renal cortex of mAb 1-22-3-injected rats. Wistar rats were nephrectomized and injected without mAb 1-22-3 (Control) or with 0.5 mg mAb 1-22-3 through the tail vein seven days after nephrectomy. Thirty-five days after nephrectomy, saline (Saline) or 10^6^ DFAT cells were injected through the renal artery (DFAT ia) or tail vein (DFAT iv). Sixty-three days after nephrectomy, the left kidney was removed. Real-time quantitative PCR was performed for expression of IL-6 **(A)**, IL-10 **(B)**, IL-12β **(C)** and TGF-β1 **(D)** mRNAs. Data are the mean ± SEM (n = 6). *P <0.05 and **P <0.01 in the indicated columns. DFAT, dedifferentiated fat; mAb, monoclonal antibody; SEM, standard error of the mean; TGF- β1, transforming growth factor- β1.

mAb-1-22-3 injection increased the expression of IL-6, IL-10, IL-12β and transforming growth factor-beta 1 (TGF-β1) mRNAs in renal cortex from Wistar rats. Implantation of DFAT cells decreased the abundance of IL-10 mRNA (not significant) and resulted in a significant (*P* <0.05) reduction in the increased abundance of IL-6 and IL-12β mRNAs (Figure 9A-9C). Implantation of DFAT cells did not affect the abundance of TGF-β1 mRNA (Figure 9D).

### Effects of implantation of DFAT cells on generation of TNF-stimulated gene 6 in DFAT cells in mAb 1-22-3-injected rats

**Figure 10:**
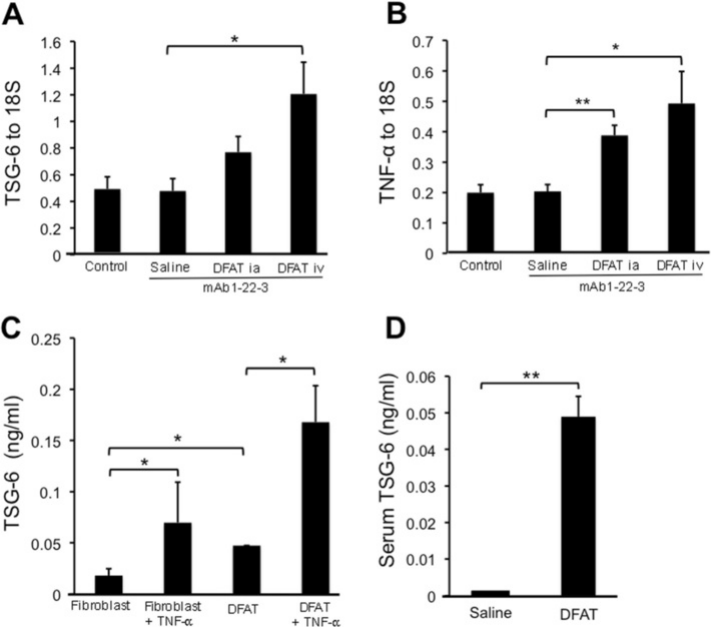
Expression of TSG-6 in kidney from mAb 1-22-3-injected rats after implantation of DFAT cells and production of TSG-6 protein in cultured DFAT cells stimulated with TNF-α. Wistar rats were nephrectomized and injected without mAb 1-22-3 (Control) or with 0.5 mg mAb 1-22-3 through the tail vein seven days after nephrectomy. Thirty-five days after nephrectomy, saline (Saline) or 10^6^ DFAT cells were injected through the renal artery (DFAT ia) or through the tail vein (DFAT iv) in rats. Sixty-three days after nephrectomy, all rats were killed and the left kidney was removed. Real-time quantitative PCR was performed for expression of TSG-6 **(A)** and TNF-α **(B)** mRNAs. **C)** Levels of TSG-6 in culture medium were determined using ELISA kits. **D)** Concentrations of TSG-6 in conditioned medium from 10^6^ fibroblast or from 10^6^ DFAT cells in culture were determined by ELISA kit. Serum levels of TSG-6 were determined in serum from Wistar rats injected with saline and 10^6^ DFAT cells. Data are the mean ± SEM (n = 6). *P <0.05 and **P <0.01 in the indicated columns. DFAT, dedifferentiated fat; mAb, monoclonal antibody; SEM, standard error of the mean; TSG-6, TNF-stimulated gene-6.

Implantation of DFAT cells through the tail vein significantly (*P* <0.05) increased the abundance of TSG-6 mRNA in renal cortex from mAb 1-22-3-injected rats (Figure 10A). Implantation of DFAT cells through the renal artery and tail vein significantly (*P* <0.05) increased the abundance of TNF-α mRNA in renal cortex from mAb 1-22-3-injected rats (Figure 10B). Figure 10C shows the production of TSG-6 protein in cultured fibroblasts and DFAT cells stimulated with TNF-α. Basal levels of TSG-6 protein in conditioned medium were significantly (*P* <0.05) higher in DFAT cells than in fibroblasts. TNF-α significantly (*P* <0.05) increased the production of TSG-6 proteins in conditioned medium from fibroblasts and DFAT cells (Figure 10C). Serum levels of TSG-6, which were significantly (*P* <0.01) increased in rats at 24 hours after implantation of DFAT cells (Figure 10D), were almost undetectable.

### Production of TNF-stimulated gene 6 from mesangial cells by transwell coculture of DFAT cells

**Figure 11:**
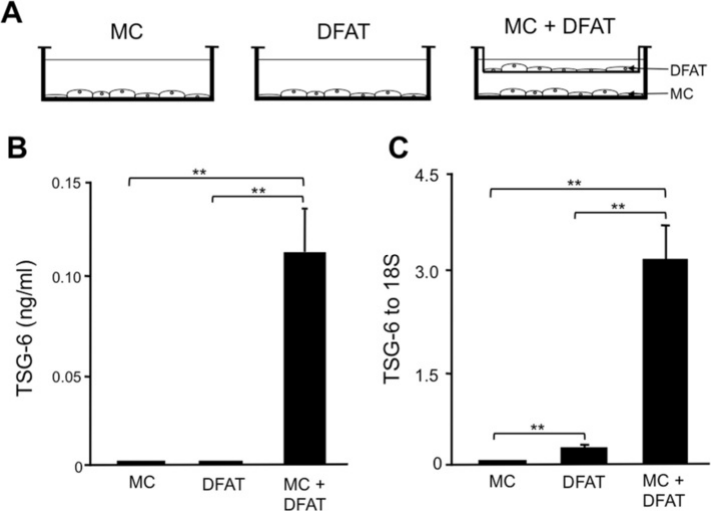
Production of TSG-6 from mesangial cells (MCs) by transwell coculture of DFAT cells. MCs from stroke-prone spontaneously hypertensive rats (SHR-SP) were plated at 4,000 cells per cm^2^ in the bottom well of a six-well transwell coculture system. DFAT cells from Wistar rats were separately plated at 1,000 cells per cm^2^ on the upper inserts of a transwell coculture system and cultured for seven days. **A)** Transwell coculture systems of DFAT cells and MCs. MC: MCs alone. DFAT: DFAT cells alone. MC + DFAT: MCs were seeded in the lower chamber and DFAT cells were seeded in the upper chamber. **B)** Levels of TSG-6 in culture medium were determined using ELISA. **C)** Expression of TSG-6 mRNA was determined by real-time RT-PCR in MCs from SHR-SP or DFAT cells. Data are the mean ± SEM (n = 6). *P <0.05 and **P <0.01 in the indicated columns. DFAT, dedifferentiated fat; SEM, standard error of the mean; TSG-6, TNF-stimulated gene-6.

To investigate whether separately placed DFAT cells can increase TSG-6 in MCs, we employed a transwell coculture system. TSG-6 concentration in conditioned medium from MCs incubated with DFAT cells was significantly (*P* <0.01) higher than in medium from MCs or DFAT cells alone (Figure 11B). The abundance of TSG-6 mRNA in MCs incubated with DFAT cells was significantly (*P* <0.01) higher than MCs or DFAT cells alone (Figure 11C), indicating that the separately placed DFAT cells increased production of TSG-6 in MCs.

### Effects of implantation of DFAT cells with TSG-6 siRNA on mAb 1-22-3-induced glomerulonephritis

**Figure 12:**
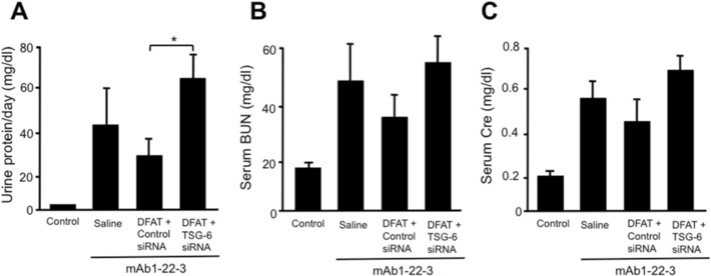
Effects of implantation of DFAT cells with TSG-6 siRNA on proteinuria and renal function in mAb 1-22-3-induced glomerulonephritis. Wistar rats were injected with 0.5 mg of mAb 1-22-3 and saline or 10^6^ DFAT cells transfected with 20 nM TSG-6 siRNA or 20 nM control siRNA through the tail vein. Twenty-one days after the neflectomy urine was collected for 24 hours and blood was sampled. Urinary protein excretion **(A)**, and serum levels of BUN **(B)** and creatinine (Cre) **(C)** were measured. Data are the mean ± SEM (n = 4). *P <0.05 in the indicated columns. Bar = 50 μm. BUN, blood urea nitrogen; DFAT, dedifferentiated fat; mAb, monoclonal antibody; SEM, standard error of the mean; TSG-6, TNF-stimulated gene-6.

We confirmed significant (*P* <0.05) suppression of TSG-6 protein concentration in conditioned medium from cultured DFAT cells transfected with 20 nM TSG-6 siRNA (Additional file 1: Figure S3A). In addition, serum concentration of TSG-6 was significantly (*P* <0.05) lower in mAb 1-22-3-injected rats implanted with DFAT cells with TSG-6 siRNA compared to rats implanted with DFAT cells with control siRNA (Additional file 1: Figure S3B). Implantation of DFAT cells with control siRNA through the tail vein reduced the increases in proteinuria. Implantation of DFAT cells with TSG-6 siRNA through the tail vein did not reduce proteinuria that was significantly (*P* <0.05) higher than proteinuria in rats implanted with DFAT cells with control siRNA. Injection of mAb 1-22-3 increased serum levels of BUN and creatinine. Systematic implantation of DFAT cells with TSG-6 siRNA through the tail vein did not decrease the increased serum levels of BUN and creatinine (Figure 12B, 12C).

**Figure 13:**
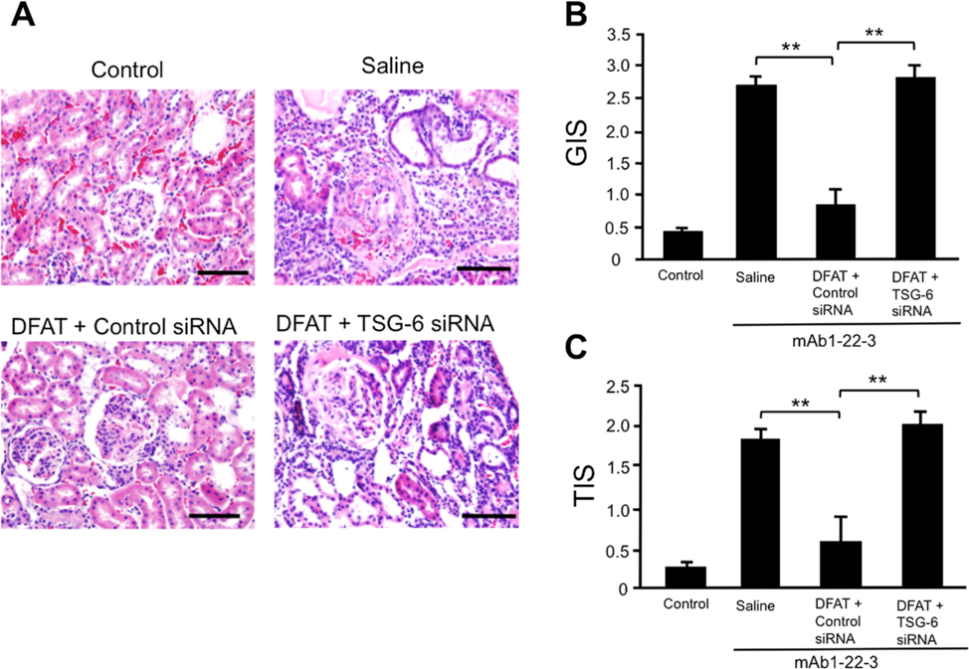
Effects of implantation of DFAT cells with TSG-6 siRNA on degeneration of renal cortex in mAb 1-22-3-induced glomerulonephritis. Wistar rats were injected with 0.5 mg of mAb 1-22-3 and saline or 10^6^ DFAT cells transfected with 20 nM TSG-6 siRNA or 20 nM control siRNA through the tail vein. Fourteen days after the injections, all rats were killed and the left kidney was removed. **A)** The paraffin sections of removed renal cortex were stained with hematoxylin and eosin (H & E). Renal cortical thickness was measured under high magnification (x400). **B)** Glomerular injury score (GIS). **C)** Tubulointerstitial injury score (TIS). Data are the mean ± SEM (n = 4). **P <0.01 in the indicated columns. Bar = 50 μm. DFAT, dedifferentiated fat; mAb, monoclonal antibody; SEM, standard error of the mean; TSG-6, TNF-stimulated gene-6.

Figure 13 shows effects of implantation of DFAT cells with TSG-6 siRNA on GIS and TIS of mAb 1-22-3-induced glomerulonephritis. Injection of mAb 1-22-3 significantly (*P* <0.01) increased the GIS and TIS. Implantation of DFAT cells with control siRNA through tail vein resulted in a significant (*P* <0.01) reduction in the increased GIS and TIS. Whereas implantation of DFAT cells with TSG-6 siRNA through tail vein did not affect the increased GIS and TIS (Figure 13).

## Discussion

As an animal model for renal diseases, anti Thy-1.1 antibody induces transient glomerulonephritis with spontaneous improvement [20], whereas mAb 1-22-3 induces glomerulonephritis with significant proteinuria and progressive mesangial injury. In this study, one injection of mAb 1-22-3 induced mesangiolysis and glomerular inflammatory cell infiltration in unilaterally nephrectomized rats. In addition, expression of Kim-1, collagen IV and fibronectin mRNAs was increased, and expression of nephrin mRNA was decreased in renal cortex from rats injected with mAb 1-22-3, indicating that mAb 1-22-3 induced acute mesangial injury as well as podocyte injury. These results suggest that mAb 1-22-3-induced glomerulonephritis is an ideal animal model for progressive immune-mediated nephropathy.

We examined the effects of implantation of DFAT cells on mAb 1-22-3-induced glomerulonephritis in rats. DFAT cells can be obtained from small amounts of adipose tissue and express HLA-A, −B, and -C, but not HLA-DR. Thus, DFAT cells may be an attractive cell source for allogenic implantation. In response to specific culture conditions, DFAT cells can differentiate into adipocytes, osteoblasts, chondrocytes, myofibroblasts, skeletal myocytes, and cardiomyocytes [21-23]. Thus, DFAT cells are considered to be a suitable cell source for regenerative medicine for the mesenchymal organs. In the present experiments, implantation of DFAT cells decreased proteinuria and improved glomerular injury as well as interstitial degeneration of the kidney in mAb 1-22-3-injected rats. Notably, the systematic implantation of DFAT cells through the tail vein was more effective in reducing proteinuria and renal degeneration than direct implantation. Labeled DFAT cells implanted through the tail vein were trapped mainly in the lung and not delivered to the kidney. These findings indicate the possibility that DFAT cells trapped in the lung release some anti-inflammatory and/or immunosuppressive substances to improve renal injury in mAb 1-22-3 injected rats.

We examined the effects of implantation of DFAT cells on adriamycin-induced glomerulonephropathy. Adriamycin induces focal segmental glomerulosclerosis with severe proteinuria with interstitial degeneration of the kidney [24]. Adriamycin induces injury by direct toxic damage to the glomerulus with subsequent tubulointerstitial injury with damages of the glomerular filtration barrier including glomerular endothelial cells, glomerular basement membrane and podocytes [25]. Subsequently, adriamycin-induced nephropathy shows severe tubulointerstitial inflammation with marked infiltration by T and B lymphocytes and macrophages [26]. However, it has been reported that adriamycin can induce structural and functional injury in immunodeficient mice without infiltration by T and B lymphocytes. These reports suggest adriamycin directly induces degeneration of glomerulus with tubulointerstitial injury without immunological mechanisms, which accompanies subsequent immunological injury of kidney with infiltration of lymphocytes. The implantation of DFAT cells did not improve the adriamycin-induced glomerular degeneration and rather increased the proteinura. It is, therefore, considered that the systematic implantation of DFAT cells mainly improved the renal degeneration of mAb 1-22-3-induced glomerulonephritis through immunosuppressive mechanisms.

It has recently been established that MSCs exert immunosuppressive effects through the inhibition of activated T cells and natural killer cells. MSCs can inhibit immune responses in non-human leukocytes in an antigen-restricted manner [27,28]. Implantation of MSCs has been reported to suppress systematic lupus erythematosus [29] and inflammatory bowel diseases [30]. The complex mechanisms underlying the immunosuppressive effects of MSCs are reported to be the increases in regulatory T cells through the induction of prostaglandin E2 (PGE2), indoleamine 2,3-dioxygenase [31], and the release of several cytokines [32] and hepatocyte growth factor (HGF) [33]. HGF has been reported to have immunosuppressive effects on reactions caused by transplantation and autoimmune diseases [34]. In the present experiments, the immunostaining of HGF was not different in renal cortex from control rats, mAb 1-22-3-injected rats injected with saline or DFAT cell-implanted rats, indicating that HGF was not involved in the improvement of renal degeneration in mAb 1-22-3 injected rats as a result of DFAT cell implantation (Additional file 1: Figure S1).

We examined the effects of DFAT cell implantation on leukocyte influx into the kidney in mAb 1-22-3-injected rats. Infiltration of ED1^+^ pan monocytes/macrophages, OX1^+^ common leukocytes and OX19^+^ pan T cells was increased in the glomeruli in kidney from mAb 1-22-3-injected rats. DFAT cell implantation significantly decreased ED1^+^, OX1^+^ and OX19^+^ cells, whereas OX38^+^ helper T cells were significantly increased. These findings indicate the suppression of macrophage infiltration into the kidney and the increase in helper T cells in mAb 1-22-3-induced glomerulonephritis might be mechanisms underlying the systematic implantation-induced immunosuppression. Furthermore, expressions of IL-6, IL-10 and IL-12β mRNAs, which were suppressed by the implantation of DFAT cells, were increased in renal cortex from mAb-1-22-3-injected rats. IL-6, IL-10 and IL-12β have been reported to be mainly produced from macrophages [35], indicating that the implantation of DFAT cells ameliorated the immunological glomerulonephritis by suppression of macrophage infiltration into the kidney with inhibition of IL-6, IL-10 and IL-12β. However, mechanisms underlying the improvement of mAb 1-22-3-induced glomerulonephritis, when DFAT cells were trapped in the lung, remain to be elucidated.

It has been demonstrated that intravenously infused MSCs were mostly trapped as emboli in the lung and that they secrete an anti-inflammatory protein, TSG-6, which decreases inflammatory responses and improves injured organs [36]. TSG-6, a 30 kDa protein, is induced by a number of signaling molecules, principally TNF-α and IL-1 and may be induced by mechanical stimuli in mesenchymal cells. It is found to be correlated with proteoglycan synthesis and aggregation [37]. Wang *et al*. [38] recently demonstrated that MSCs trapped in the lung produce TSG-6 after intravenous injection, which induced anti-inflammatory and immunosuppressive effects. Interestingly, they also demonstrated that intravenous injection of conditioned medium of cultured MSCs increased serum levels of TSG-6 as well as tissue levels of TSG-6 in injured peritoneum [39]. In the present experiments, the systematic implantation of DFAT cells increased serum levels of TSG-6 as well as the expression of TSG-6 in the kidney of mAb 1-22-3-injected rats. In addition, the systematic implantation increased expression of TNF-α mRNA in renal cortex from mAb 1-22-3-injected rats, which may stimulate the expression of TSG-6 in renal cortex. Moreover, we found that production of TSG-6 protein was higher in DFAT cells than in fibroblasts and that the implantation of DFAT cells increased serum levels of TSG-6 protein in rats. These findings suggest that the implanted DFAT cells abundantly produced TSG-6, which ameliorated the mAb 1-22-3-induced glomerulonephritis.

To investigate whether separately placed DFAT cells can increase TSG-6 in MCs, we employed a transwell coculture system. Production of TSG-6 in conditioned medium from MCs with DFAT cells was higher than MCs or DFAT cells alone. Expression of TSG-6 mRNA in MCs cocultured with DFAT cells was higher than in MCs or DFAT cells alone, indicating that the separately placed DFAT cells increased TSG-6 in MCs. Recently, Roddy *et al*. [40] demonstrated that MSCs are activated by proinflammatory signals to introduce two phases: 1) the activated MSCs secrete PGE2, which drives resident macrophages of type 1 proinflammatory phenotype toward type 2 anti-inflammatory phenotype macrophages; and 2) the activated MSCs secrete TSG-6, which interacts with CD44 on resident macrophages to decrease toll-like receptor 2. TSG-6 from MSCs inhibits the initial activation of resident macrophages by modulating toll-like receptor 2/CD44/NF-kB signaling and thereby decreases the phase II inflammatory response. These findings are identical to our own in that the systematic implantation of DFAT cells reduced macrophage infiltration into the kidney with decreases the expression of IL-6, IL-10 and IL-12β mRNAs in mAb 1-22-3-injected rats.

In order to explore the contribution of TSG-6 in the amelioration of the mAb 1-22-3-induced glomerulonephritis with implantation of DFAT cells, we examined effects of implantation of DFAT cells transfected with TSG-6 siRNA. Implantation of DFAT cells with control siRNA through the tail vein reduced the increases in proteinuria. Systematic implantation of DFAT cells with TSG-6 siRNA through the tail vein did not reduce proteinuria, increases in serum BUN and creatinine and renal degeneration in the mAb 1-22-3-injected rats. These results suggest that the DFAT cells trapped in the lung produced TSG-6 in serum as well as in the kidney, which improved the renal degeneration and the renal dysfunction in rats with the mAb 1-22-3.

## Conclusions

Systematic implantation of DFAT cells effectively ameliorated mAb 1-22-3-induced glomerulonephritis through immunosuppressive effects accompanied by the suppression of macrophage infiltration and expression of IL-6, IL-10 and IL-12β, and increased production of serum and renal TSG-6 that improved the mAb 1-22-3-induced renal degeneration by the immunosuppressive effects of TSG-6. Thus DFAT cells will be a suitable cell source for the treatment of immunological progressive renal diseases.

## Additional file

Additional file 1: Figure S1. Effects of implantation of DFAT cells on population of regulatory T cells in mAb 1-22-3-injected rats. **Figure S2.** Effects of implantation of DFAT cells on expression of HGF in kidney from mAb 1-22-3-injected rats. **Figure S3.** Suppressions of TSG-6 in DFAT cells and in serum with TSG-6 siRNA.

## Abbreviations

ASCs: adipose-derived stromal cells
BUN: blood urea nitrogen
DFAT: dedifferentiated fat
ELISA: enzyme-linked immunosorbent assay
GIS: glomerular injury score
HGF: hepatocyte growth factor
H & E: hematoxylin and eosin
IL: interleukin
mAb: monoclonal antibody
Kim: kidney injury molecule
MCs: mesangial cells
MSCs: mesenchymal stem cells
PBS: phosphate-buffered saline
SHR-SP: stroke prone spontaneous hypertensive
siRNA: small interfering RNA
TGF: transforming growth factor
TIS: tubulointerstitial injury score
TNF: tumor necrosis factor
TSG: TNF-stimulated gene
