# Supplementary material for: Systematic implantation of dedifferentiated fat cells ameliorated monoclonal antibody 1-22-3-induced glomerulonephritis by immunosuppression with increases in TNF-stimulated gene 6
Source: Stem Cell Res Ther. 2015 Apr 16;6(1):80. doi: 10.1186/s13287-015-0069-2 (PMC4455708; doi:10.1186/s13287-015-0069-2)
Supplement: Additional file 1: Figure S1. — Effects of implantation of DFAT cells on population of regulatory T cells in mAb 1-22-3-injected rats. Figure S2. Effects of implantation of DFAT cells on expression of HGF in kidney from mAb 1-22-3-injected rats. Figure S3. Suppressions of TSG-6 in DFAT cells and in serum with TSG-6 siRNA. [file 13287_2015_69_MOESM1_ESM.pdf]

SUPPLEMENTARY MATERIALS

Supplementary Figure 1.

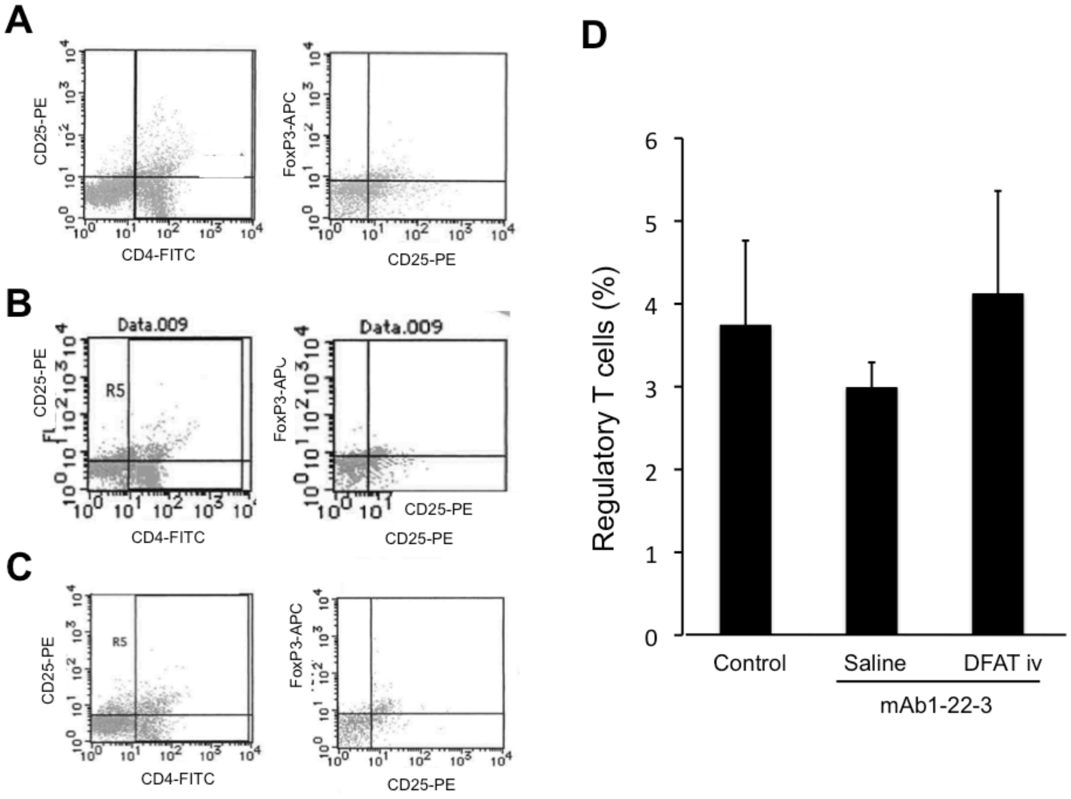

Effects of implantation of DFAT cells on population of regulatory T cells in mAb 1-22-3-injected rats. Spleen was removed and homogenized 4 weeks after implantation of 10^6 of DFAT cells in rats injected with mAb 1-22-3. Cells from spleen were labelled with fluorogenic antibodies, CD4-APC-Cy7, CD25-AlexaFluor®647, FOXP3-PerCP5.5 to evaluate the proportion of CD4$^+$ CD25$^+$ FOXP3$^+$ regulatory T cells. Cells were fixed and permeabilized with a FOXP3 Staining Buffer Set according to manufacturer's instructions and including the blocking step with 2% rat serum. Flow cytometry was performed with FACSAria and data were analyzed using the FlowJo 7.6.5 software. The number of CD4$^+$ CD25$^+$ FOXP3$^+$ regulatory T cells in spleen was lower in rats injected with mAb 1-22-3 than in normal rats. There was no significant difference between control rats and mAb 1-22-3-injected rats with saline and DFAT cells implanted through the tail vein in the number of CD4$^+$ CD25$^+$ FOXP3$^+$ regulatory T cells.

Supplementary Figure 2.

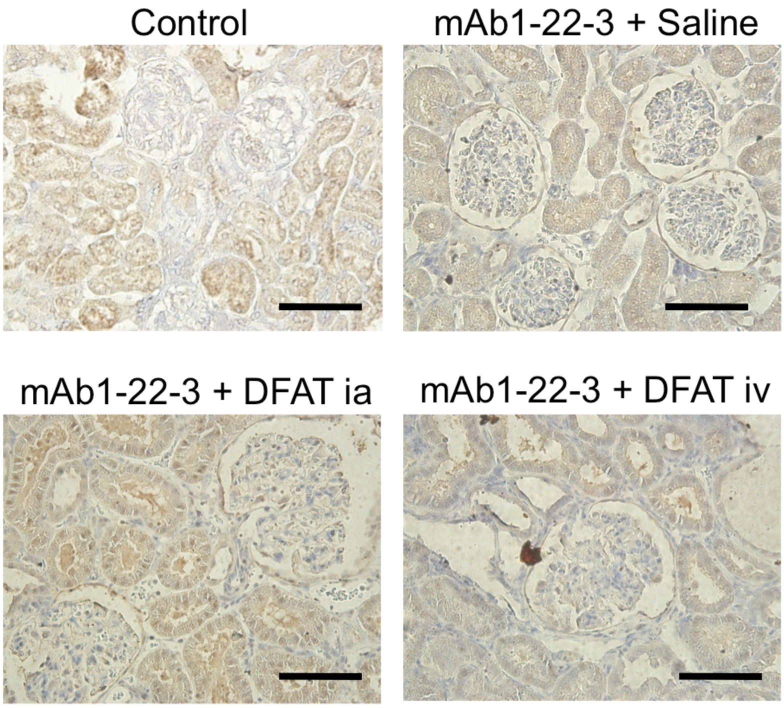

Effects of implantation of DFAT cells on expression of HGF in kidney from mAb 1-22-3-injected rats. Wistar rats were nephrectomized and injected without mAb 1-22-3 (Control) or with 0.5 mg of mAb 1-22-3 through the tail vein 7 days after the nephrectomy. Thirty-five days after the nephrectomy saline (Saline) or 10^6 of DFAT cells were injected through renal the artery (DFAT ia) or tail vein (DFAT iv). Sixty-three days after the nephrectomy, the left kidney was removed. The paraffin sections of removed renal cortex were stained with HGF antibody (R & D Systems). Horseradish peroxidase labeling was detected using a peroxide substrate solution with diaminobenzidine and 0.01% H_2O_2. Immunohistochemistry staining shows HGF was mainly expressed in the proximal tubulus. HGF in renal cortex from control rats did not differ between saline or DFAT cell-implanted mAb 1-22-3 injected rats. Bar = 50 μm.

Supplementary Figure 3.

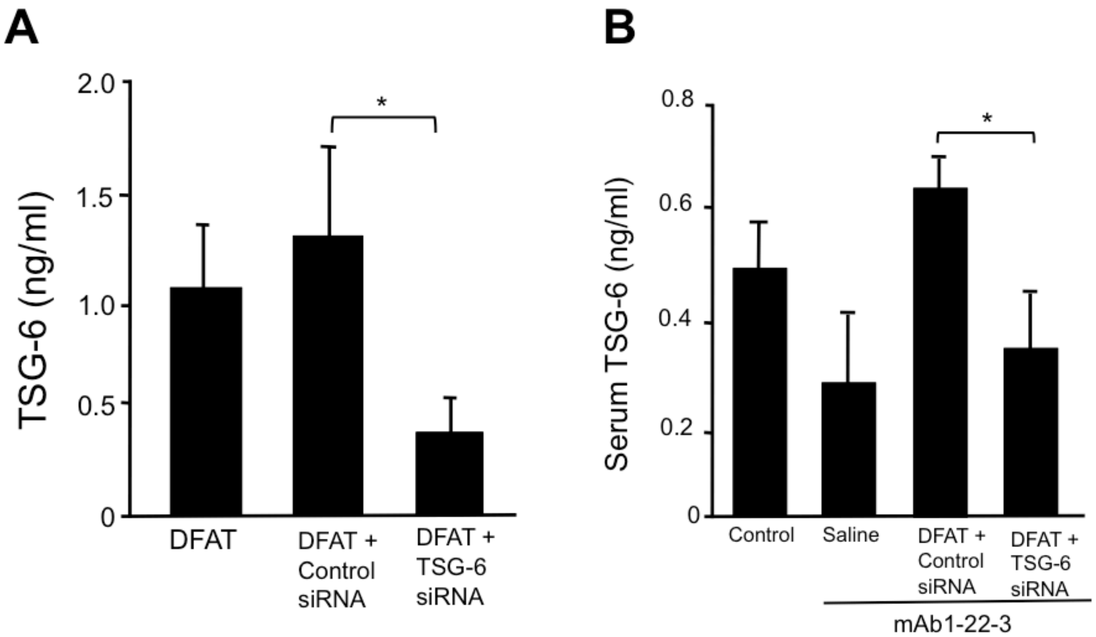

Suppressions of TSG-6 in DFAT cells and in serum with TSG-6 siRNA. (A) To confirm sufficient inhibition of expression of TSG-6 protein, we performed ELISA analysis for siRNA transfected DFAT cells at 24 hours after transfection. DFAT cells (2 x 10^5 cells) from Wistar rats were transfected with rat TSG-6 siRNA or control siRNA in siRNA Transfection Medium. (B) In male Wistar rats weighing 250 g, the right kidney was nephrectomized. Rats were injected with 0.5 mg of mAb 1-22-3 and 10^6 of DFAT cells transfected with 20 nM TSG-6 siRNA or 20 nM control siRNA through the tail vein. Serum TSG-6 concentrations were measured by ELISA analysis. Data are the mean ± SEM (n=4). **P* <0.05 in the indicated columns.
